# Isothermal nucleic acid amplification assays for the detection of porcine stool-associated RNA virus

**DOI:** 10.1038/s41598-025-22146-4

**Published:** 2025-10-31

**Authors:** Sarshti Kaushik, Sushila Maan, Kanisht Batra, Swati Sindhu, Vijay Kadian, Aman Kumar

**Affiliations:** Department of Animal Biotechnology, Lala Lajpat Rai University of Veterinary and Animal Sciences, Hisar, 125004 Haryana India

**Keywords:** Recombinase polymerase amplification, RPA, Polymerase spiral reaction, PSR, Porcine stool associated RNA virus, Posavirus, Isothermal assays, Picornavirus, Biological techniques, Biotechnology, Microbiology, Molecular biology

## Abstract

Pigs are a vital component of agricultural economies and a major source of livestock worldwide. The Porcine Stool-Associated virus (Posavirus), a newly identified member of the *Picornavirales* order, has been associated with enteric infections in swine. Recombinase Polymerase Amplification (RPA) and Polymerase Spiral Reaction (PSR), two isothermal amplification methods, were developed and optimized in this study to identify the posavirus in pig stool samples quickly and effectively. Primers that target the posavirus’s polyprotein region were designed for both RPA and PSR assays, and reaction parameters were optimized. Sensitivity assessments revealed that the RPA assay had a detection limit of 5.34 × 10^6^copies, while the PSR assay has higher sensitivity at 6.5 × 10^3^copies. Both assays showed high specificity for the posavirus, with no cross-reactivity. An evaluation of 132 field samples revealed that only three samples were positive for posavirus, highlighting the need for continued surveillance. This study reported the successfully development and optimisation of RPA and PSR assays as dependable and easily accessible diagnostic methods for posavirus detection. Their speed, sensitivity, and specificity make them adapted for use in a range of field and laboratory scenarios.

**Supplementary Information:**

The online version contains supplementary material available at 10.1038/s41598-025-22146-4.

## Introduction

Pigs are among the most common livestock animals globally, raised under a wide variety of farming conditions. They can flourish in highly populated, intensive, industrialized environments, often coexisting with people, other farmed animals, or wildlife, and they can adapt to situations that are close to natural or even wild. These diverse environments make pigs an ideal host for rapid viral genome alterations through recombination and reassortment, which may lead to the emergence of new viruses that are dangerous to animal and human health. Additionally, diarrhea significantly impacts the swine industry’s profitability^1^. Porcine epidemic diarrhea virus (PEDV), porcine transmissible gastroenteritis virus (TGEV), porcine delta coronavirus (PDCoV), and porcine rotavirus (PoRV) are among the several porcine viruses that can cause diarrhea in pigs^2^. Pig diarrhea may also be caused by enteroviruses belonging to the *Astroviridae*,* Caliciviridae*,* Coronaviridae*, and *Picornaviridae* families^3^.

The order *Picornavirales* possess a wide variety of RNA viruses that infect multiple hosts and is divided into five families: *Dicistroviridae*, *Iflaviridae*, *Secoviridae*, *Picornaviridae*, and *Marnaviridae*^4^. Among these, the Porcine Stool-Associated RNA virus (Posavirus), a single-stranded, positive-sense RNA virus, is a new member of the order *Picornavirale*. Members of this order despite their considerable diversity, share several key features, including a single-stranded RNA genome and conserved replication domains (Hel-Pro-Pol) that encode essential enzymatic functions such as helicase, protease, and RNA-dependent RNA polymerase^5^. Specific viral proteases digest polyproteins, and genome length vary from 7.2 to 9.8 kilobases. While most viruses within the *Picornavirales* order possess monopartite genomes, certain members of the *Secoviridae* family exhibit segmented genomes^5^.

Since its first discovery in 2011 in American piglets with diarrhea, the posavirus has been found in both healthy piglets and adult pigs^6,7^. With detections in South Africa, Japan, South Korea, Belgium, Germany, and China, this virus has been reported worldwide^8–12^. Posavirus 1 and posavirus 3 are the most common of the twelve different lineages that have been found, referred to as posavirus 1 through posavirus 12^9,11^. While posavirus 3 has mostly been seen in the US and South Korea, posavirus 1 has been found in several country^6,7,9^. Previous studies have reported the presence of posavirus in 21–40% of pig fecal and barn environmental samples, typically in combination with other enteric viruses in both healthy and diarrheic pigs^13,14^. Its association with diarrhea remains unclear, and current evidence indicates it is unlikely to be a primary pathogen in swine. Porcine posavirus has been identified in pig populations across several countries, highlighting its widespread distribution in both healthy and diarrheic animals including India^15^. There is currently no data or published research that quantifies the economic burden of posavirus neither in India nor abroad. It’s a newly recognized virus, and both its prevalence and impact remain largely unstudied and is an important gap for future research. At present, no commercial vaccines or specific antiviral therapies are available, and control depends mainly on supportive care and strict biosecurity measures^16^.

A crucial component of Indian agriculture, swine production greatly boosts both the country’s economy and farmers’ livelihoods. Concerns over the posavirus’s involvement in enteric disease outbreaks have been raised by recent research showing that it is present in pig populations. Like other enteric viruses, the posavirus is thought to spread through fecal-oral pathways, while epidemiology in India is still being studied. The virus can spread more widely throughout pig populations due to factors like close-contact farming, poor cleanliness, and insufficient biosecurity measures. Given India’s difficulties managing livestock health, it is essential to comprehend and keep an eye on the posavirus in order to maintain swine productivity and health.

In addition to infecting gut commensals, the porcine posavirus can potentially come from environmental or dietary sources. Some strains of the posavirus and the parasite *Ascaris suum* share similar sequences, according to BLAST studies^6,17^. The presence of the virus in the digestive tract complicates diagnostics and necessitates reliable detection methods. Traditional diagnostic methods like virus isolation and serological testing are no longer effective due to their limitations in terms of speed, sensitivity, and scalability. Despite being more precise and effective, molecular techniques like RT-PCR and qPCR are usually impractical in resource-constrained settings. However, novel isothermal amplification technologies such as Recombinase Polymerase Amplification (RPA) and Polymerase Spiral Reaction (PSR) provide good alternatives that enable simpler and faster diagnoses in less complex testing environments and help implement targeted control measures. RPA is an isothermal nucleic acid amplification technique in which recombinase-bound primers invade homologous sites on dsDNA to form a D-loop. The displaced strand is stabilized by single-stranded binding (SSB) proteins, while a strand-displacing polymerase extends from the primer. ATP facilitates the dynamic assembly of the recombinase–primer complex, and mediator proteins such as UvsY enhance primer loading. This cycle repeats with successive primer sets, resulting in rapid DNA amplification at an isothermal temperature of approximately 39 °C^18^. PSR is another isothermal amplification method that shares similar principles with RPA. It employs specially designed primers and a DNA polymerase with strand-displacement activity. Once primers anneal to the target sequence, the polymerase continuously synthesizes new DNA strands while displacing existing ones, generating a spiral-like chain reaction of amplification. Owing to its single-temperature operation, PSR is simple, rapid, and particularly well suited for application in resource-limited settings^19^.

Although virus isolation has not yet been successful for posavirus due to the lack of a suitable cell culture system, its identification has been reliably achieved through metagenomic next-generation sequencing. In our earlier metagenomic NGS-based investigation of diarrheic pig fecal samples in Haryana, India, posavirus was identified for the first time in the country, confirming its circulation in local pig populations^15^. These genomic data not only established the presence of posavirus in India but also provided the sequence information that enabled the design of primers for the present study. Building upon this discovery, we aimed to develop rapid, field-deployable isothermal assays for posavirus detection, thereby facilitating future epidemiological and economic studies.

## Materials and methods

### Sample details and viral RNA extraction

A total of 132 archived samples, collected from various organized and unorganized piggery farms in Haryana between March and October 2021, were included in this study. The samples, which came from pigs of all age groups, consisted of both fecal and nasal swabs. Fecal samples were collected in stool collection vials, while nasal samples were taken using sterile swabs and stored at −20 °C until further analysis. The samples were then dissolved in PBS (10% w/v), and debris was removed by centrifugation at 10,000 xg for 10 min. The supernatant was collected in separate microcentrifuge tubes and stored at −20 °C for further processing. Viral RNA extraction was performed using the Trizol method in combination with QIAmp viral RNA kit (Qiagen). Briefly, swab samples were resuspended in 400 µL of PBS. To this 400 µL of Trizol reagent was added and mixture was vortexed. To this 200 µL of chloroform was added and contents were centrifuged at 12,000 rpm for 10 min. The aqueous phase containing nucleic acid was taken and to this an equal volume of isopropanol was added. This mixture was then transferred to column that come with QIAamp Viral RNA Kit (Qiagen). The columns were centrifuged at 10,000 rpm for 1 min. The flow through was discarded. The columns were then washed with 500 µL of AW1 solution and 500 µL of AW2 solution successively by centrifugation at 12,000 rpm for 1 min. The viral RNA was eluted in 30 µL of nuclease free water. The extracted RNA was quantified, and its purity was assessed using a Nanodrop 200 (Thermo Fisher Scientific Inc.).

### Designing of primers

Primers for the RPA and PSR assays were designed based on posavirus genomic sequences obtained from our earlier NGS-based identification of the virus in Indian pigs^15^. The RPA primers for posavirus were designed in-house to target the polyprotein region of the genome. The forward primer sequence is 5’-ACATGGTCTTGAGTATTATCA-3’, and the reverse primer sequence is 5’-AGTAGCAGTAACATTTGATTC-3’, producing an amplicon of 163 bp These primers were synthesized by Sigma-Aldrich Chemical Pvt. Ltd. Bangalore, India and purified using HPLC.

PSR primers were also designed to target the polyprotein region of the genome. The forward primer sequence is 5’-ttgaaattgaaaccattTGTGGTGATGATCATGT-3’, and the reverse primer sequence is 5’-ttaccaaagttaaagtttTAATCTTCAATCTCAGCAT-3’, yielding an amplicon of 164 bp. An adapter oligonucleotide of exogenous origin was added at the 5’ end of the primers, with a melting temperature (Tm) 5 °C lower than the primer sequence. These primers were synthesized by Sigma-Aldrich Chemical Pvt. Ltd. Bangalore, India and desalted.

### Preparation of cDNA

Viral RNA was reverse transcribed into cDNA using the Promega GoScript™ cDNA kit, following the manufacturer’s standard protocol. The cDNA synthesis was carried out in a 20 µL reaction with up to 5 µg of total RNA. The reaction mixture consisted of 4 µL of 5X reaction buffer, 0.5 µg of random hexamer, 1 µL of 10 mM dNTPs (0.5 mM of each), 20 U of RNase inhibitor, 1.5 mM MgCl_2_, and 1 µL of reverse transcriptase enzyme. The primer-template pre-mixes were denatured at 70 °C for 5 min and then snap chilled on ice. The remaining components were added, and the reverse transcription was performed at 25 °C for 5 min, followed by incubation at 42 °C for 1 h and a final incubation at 72 °C for 15 min.

### Gene construct

Two different regions of polyprotein region of the posavirus genome which were cloned into the pUC57 vector were sourced from Bio Basic Inc. Ontario, Canada. The synthetic recombinant plasmid having gene 2 (spanning 6571–6970 bp of posavirus genome) was used as positive control for RPA assay development while synthetic recombinant plasmid having gene 1 (spanning 6193–6580 bp of posavirus genome) was used as positive control for PSR assay development. To amplify these plasmids, there were individually transformed into *E. coli* DH5 alpha cells. Plasmid extraction was performed using the Zymopure Mini Prep Kit according to the manufacturer’s instructions. The concentration and quality of the extracted plasmid DNA were measured using a Nanodrop 2000.

### RPA assay optimization for posavirus

The RPA assay was conducted using the TwistAmp^™^ Basic Kit (TwistDx, Inc., Maidenhead, United Kingdom; catalog number TABAS03KIT). The kit contains rehydration buffer, a pellet (which includes enzymes), and magnesium acetate. Following the manufacturer’s instructions, the pellet was dissolved in the rehydration buffer. A recombinant plasmid, used as a positive control, was combined with specific primers. The RPA reaction mixture was prepared to a total volume of 50 µL with the following components: 2.4 µL each of forward and reverse RPA primers (10 µM), 29.5 µL of rehydration buffer, 1.32 µL of NFW, 12 µL of DNA template, and 2.48 µL of magnesium acetate to initiate the reaction. The RPA conditions were optimized by varying parameters such as temperature, time, forward and reverse primer concentrations, and magnesium acetate concentration. To determine the optimal reaction temperature, the assay was tested at 35 °C, 37 °C, 39 °C, 41 °C, 43 °C, and 45 °C for 20 min, using a known concentration of plasmid DNA as the template. The optimal reaction time was determined by testing durations of 5, 10, 15, 20, 25, and 30 min. The optimal primer concentration range (0.24–0.96 µM for both forward and reverse primers) was identified using the checkerboard titration method, with a total of 16 combinations tested. Finally, magnesium acetate concentrations of 10 mM, 12 mM, 14 mM, 16 mM, 18 mM, and 20 mM were tested to achieve maximum assay efficiency.

### PSR assay optimization for posavirus

PSR of the posavirus genome was carried out using Isothermal Amplification Buffer (New England Biolabs, NEB), Betaine (Sigma-Aldrich Chemical Pvt. Ltd. Bangalore, India), and *Bst* polymerase enzyme (New England Biolabs). A plasmid DNA was used as a positive control, and in-house designed primers were employed to optimize the PSR conditions for the posavirus. The PSR reaction mixture was prepared to a final volume of 25 µL, consisting of 2.5 µL of 10X Isothermal buffer, 2 µL of 50 mM MgSO4, 5 µL of 5 M Betain, 1 µL of 10 mM dNTP, 5 µL each of forward and reverse primers (10 µM), 0.5 µL of *Bst* DNA polymerase, template DNA, and nuclease-free water (NFW) to achieve the final volume. Optimization of PSR conditions for posavirus was conducted by varying parameters such as temperature, time, and primer concentrations. The optimal reaction temperature was determined by testing at 61 °C, 62 °C, 63 °C, 64 °C, 65 °C, 66 °C, 67 °C, and 68 °C for 2 h, using a known concentration of plasmid DNA as the template. The optimal reaction time was determined by performing the reaction for 1, 1.5, 2, and 2.5 h. The optimal concentration range for both forward (1–4 µM) and reverse primers (1–4 µM) was established through the checkerboard titration method, testing a total of 16 different combinations.

### Evaluation of limit of detection of assays

The analytical sensitivity of the RPA and PSR assays was evaluated using a ten-fold serial dilution of the positive control (ranging from 10^−1^ to 10^–10^). The sensitivity of the assays was determined by calculating the plasmid copy number detected by the assay. The Limit of Detection (LOD), or analytical sensitivity, was expressed in terms of the copy number. The copy number was calculated based on the quantified DNA concentration using the following formula:

$${\rm Copy\: number = [AxNo]/ [length (plasmid+ insert size) \times1\times10^{9}\times660]}$$

In this formula, A represents the DNA concentration in ng/µL.

No. is Avogadro’s number (6.022 × 10^23^).

The average weight of a nucleotide base pair (bp) is assumed to be 660 Daltons, and the number of template copies in the sample can be estimated by multiplying the DNA concentration by 1 × 10^9^ (conversion factor for ng).

### Evaluation of limit of detection of assays using DNA binding dye

PicoGreen is a fluorescent dye known for its high specificity and sensitivity in detecting dsDNA, making it more effective than many other fluorescent probes^20^. It has been effectively used to dye cellular chromosomes, measure dsDNA in solution, and perform quantitative PCR (qPCR) tests^21^. Apart from dsDNA, PicoGreen exhibits exceptional sensitivity in detecting extremely low amounts of nucleic acids, such as hybridized single-stranded nucleic acids, siRNAs^38^, and miRNA mimics. Though useful for quantification, its high affinity for dsDNA renders it inappropriate for use in PCR processes, where DNA quantities vary dynamically over amplification cycles^22^. Here, the DNA binding dye PicoGreen^®^ (Invitrogen) was used for colorimetric detection of amplicons. The RPA and PSR reaction mixtures were prepared using serially diluted plasmid and incubated under optimal temperature and time conditions. Sensitivity was then assessed using PicoGreen^®^dye. A total of 1 µL of PicoGreen^®^ dye (diluted 1:10) was added to 10 µL of the RPA and PSR reaction products, and fluorescence was observed using a UV transilluminator.

### Analytical specificity of assays

The analytical specificity of any assay refers to the ability of the assay to accurately identify and measure a specific target. To assess specificity, the assay was tested against viruses related to the target pathogen. The analytical specificity assessment was conducted by testing the assay against related viral pathogens of pigs. This was done using positive controls for Porcine Enterovirus-G (PEV-G), Porcine Sapelovirus (PSV), Porcine Parvovirus (PPV), Porcine Circovirus (PCV), and Classical Swine Fever Virus (CSFV), which were available in our laboratory. The detection of these viruses was carried out using each of the developed assays, and the results were subsequently visualized on a 2.5% agarose gel.

### Evaluation of developed assays

The developed assay was evaluated using field samples, including 132 faecal samples and nasal swabs collected from both diseased and healthy pigs in organized and unorganized farms in Haryana (29.0588° N, 76.0856° E), India. These samples were screened using the developed RPA and PSR assays (as described in material and methods section on optimization of RPA and PSR assay), as well as conventional PCR. For conventional PCR, RPA primers at a concentration of 0.48 µM for both forward and reverse primers were used. cDNA prepared from field samples (as described in material and methods section on preparation of cDNA) served as the template for the PCR reaction. The thermal conditions of PCR were finally optimized as initial denaturation at 95 °C for 2 min followed by 40 cycles of denaturation at 95 °C for 1 min, annealing at 50 °C (RPA primers)/56 °C (PSR primers) for 30 s and extension at 72 °C for 45 s and final elongation at 72 °C for 10 min.

### Ethics considerations

The study methods were carried out following ARRIVE guidelines (https://arriveguidelines.org). The ethical committee approval was taken prior to sample collection. The animal experiment was conducted as per guidelines approved by the Institutional Animal Ethics Committee (IAEC), registered as 1669/GO/ReBiBt/S/12/CPCSEA dated 6.12.2012. Before taking swab samples from the animals, a brief explanation of the study and a discussion of it were given to the owners of the animals, and each owner provided signed informed consent. Swabs were collected in accordance with the procedures for research and testing animals.

## Results

### Optimization of reaction parameters for RPA assay

The reaction conditions for the RPA were optimized at 45 °C for 20 min. While RPA was effective at temperatures of 35 °C, 37 °C, 39 °C, and 41 °C, the intensity of the amplified product bands indicated that 45 °C was the optimal temperature (Figure S1). To further refine the reaction time, incubation was tested at various intervals: 5, 10, 15, 20, 25, and 30 min at 45 °C. Amplification was detectable starting from the 5-minute mark, but the most pronounced and clearly visible signal was achieved at 20 min. Therefore, a reaction time of 20 min was determined to be optimal for the RPA process (Figure S2).The optimal primer concentration was determined using a combination of 0.96 µM for the forward primer and 0.72 µM for the reverse primer, which resulted in the highest amplification; thus, this combination was designated as optimal (Figure S3). Additionally, the concentration of magnesium acetate (MgOAc) was optimized within a range of 10 mM to 20 mM, with the best amplification observed at 16 mM (Figure S4). Consequently, a magnesium acetate concentration of 16 mM was established as the optimal condition for the RPA assay targeting the posavirus. All the reaction parameters were optimized three times for achieving reproducible results.

### Optimization of reaction parameters for PSR assay

To optimize the temperature for the PSR reaction, the mixture was incubated at temperatures ranging from 61 °C to 68 °C, with 1 °C increments. Although PSR was effective across all tested temperatures, maximum amplification was achieved at 66 °C, establishing this temperature as optimal for the PSR assay of the posavirus (Figure S5). For time optimization, the PSR reaction mixture was incubated at the standardized temperature of 66 °C for varying time intervals. Amplification was first detectable after 1 h, but the highest levels of amplification were observed at 2.5 h. Therefore, a time period of 2.5 h was deemed optimal for the PSR of the posavirus (Figure S6).The optimal concentrations of both forward and reverse primers were determined using a checkerboard method. In this approach, 16 combinations of the primers were tested, maintaining the concentration of one variable (the reverse primer) constant while incrementally increasing the concentration of the other variable (the forward primer) by 5 µM. The combination of 4 µM for the forward primer and 3 µM for the reverse primer yielded the highest amplification, and this combination was thus established as optimal for the PSR assay of the posavirus (Figure S7). All the reaction parameters were optimized three times for achieving reproducible results.

### Analytical sensitivity of assays

**Fig. 1:**
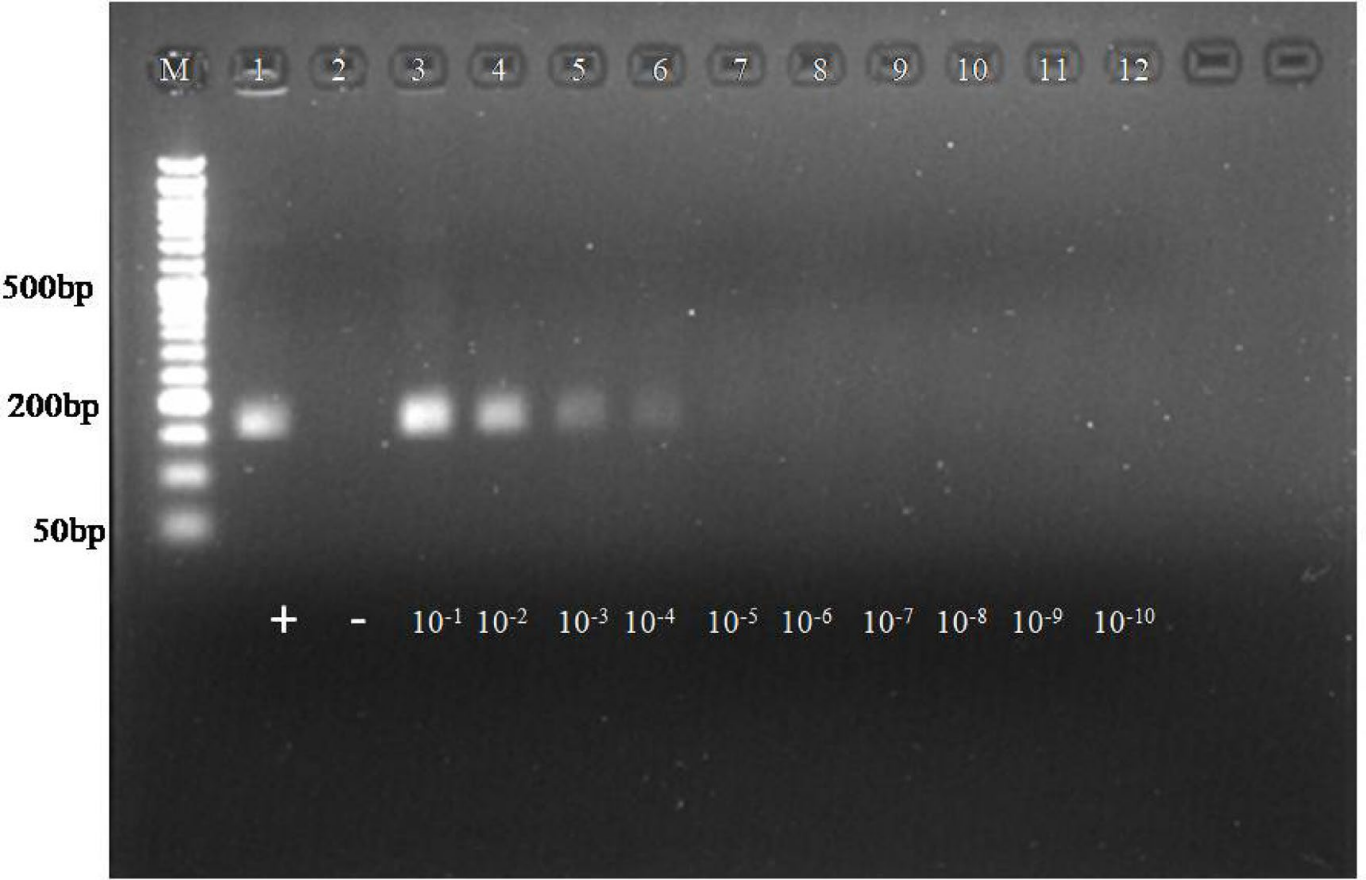
Analytical sensitivity of RPA reaction. Lane M: 50 bp ladder, L1: positive control, L2: NTC, L3-L12: Serial 10-fold dilution of posavirus plasmid DNA (10^−1^ to 10^−10^). The amplification observed upto 4^th^ dilution which corresponds to 5.34 × 10^6^ copies.

The analytical sensitivity of both assays for the posavirus was evaluated using ten-fold serial dilutions of the positive control (plasmid DNA), ranging from 10⁻¹ to 10⁻¹⁰. In the RPA assay, amplicons were clearly detectable up to the 4^th^ dilution, corresponding to approximately 5.34 × 10⁶ copies. No amplification was observed in the non-template control (Fig. 1). For comparative purposes, the same plasmid DNA dilutions were utilized in a PCR reaction. The conventional PCR assay detected plasmid DNA up to the 8^th^ dilution, whereas the developed RPA isothermal assay was limited to detection at the 4^th^ dilution (Figure S8).

**Fig. 2:**
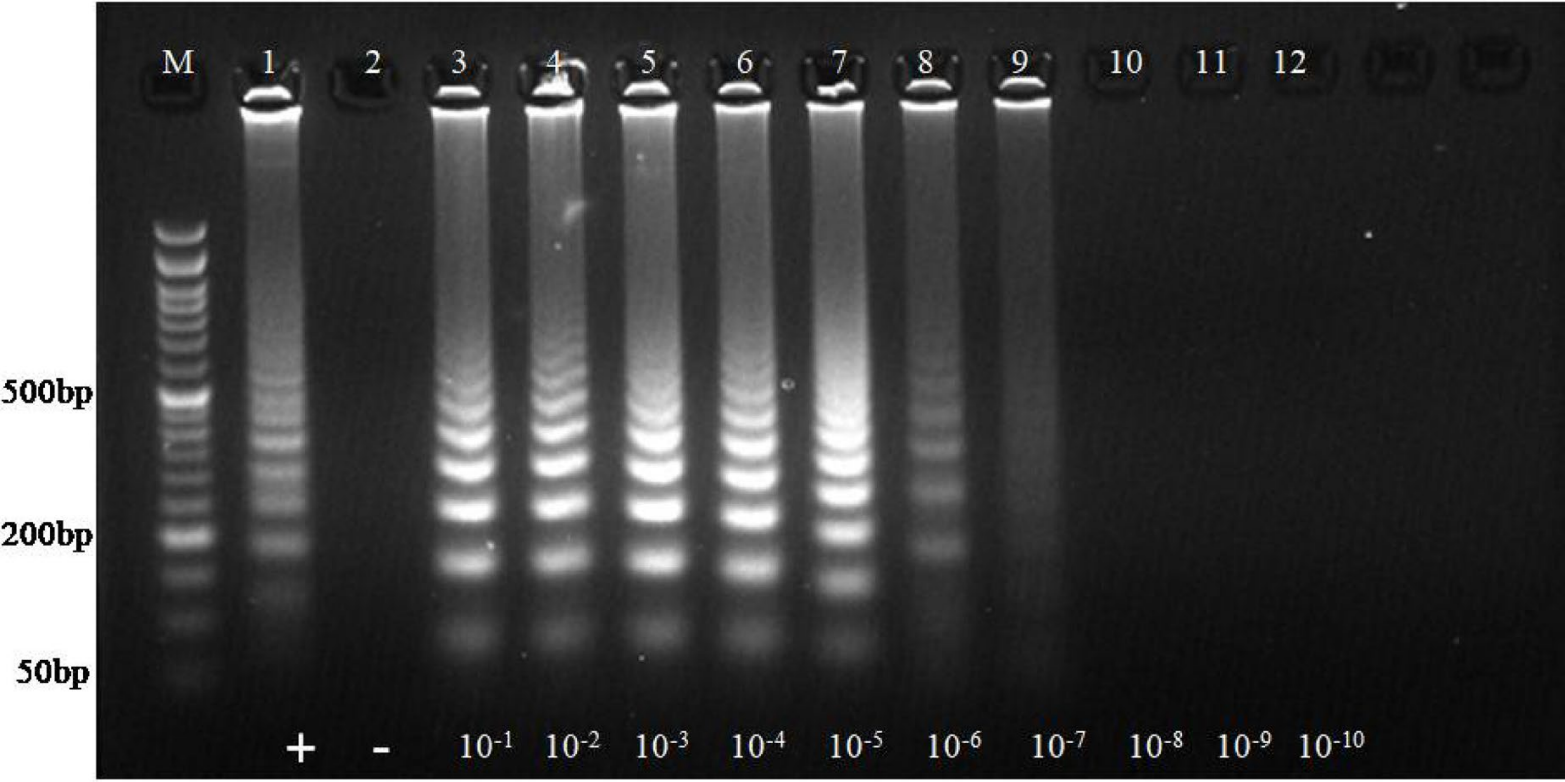
Analytical sensitivity of PSR reaction. Lane M: 50 bp ladder, L11: NTC, L1-L10: Serial 10-fold dilution of posavirus plasmid DNA (10^−1^ to 10^−10^). The amplicons are detectable upto 7^th^ dilution which corresponds to 6.5 × 10^3^ copies in agarose gel electrophoresis (AGE).

In the PSR assay, amplicons were detectable up to the 7^th^ dilution, which corresponded to approximately 6.5 × 10³ copies, with no amplification in the non-template control (Fig. 2). The same plasmid DNA dilutions were employed in the PCR reaction for comparison. The conventional PCR assay achieved amplification up to the 5th dilution, indicating that it is less sensitive than the developed isothermal PSR assay (Figure S9).

### Visual detection of both assays

Both assays were visually assessed using a series of plasmid DNA dilutions ranging from 10⁻¹ to 10⁻¹⁰, employing picogreen dye for detection. In this system, negative results appeared colourless, while positive results exhibited distinct green fluorescence.

**Fig. 3:**
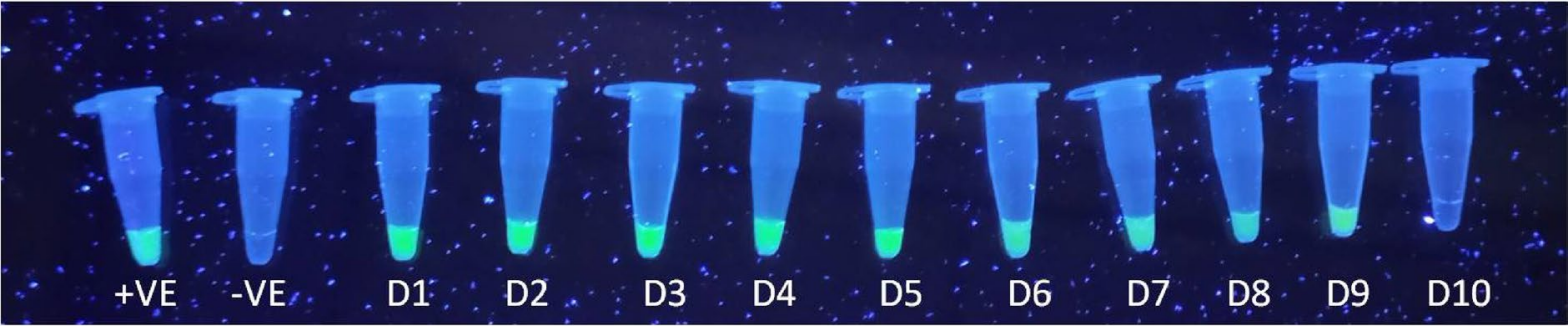
**Visual detection of posavirus RPA amplicon with different dilutions of plasmid DNA (10**^**−1**^ **to 10**^**–10**^**) using PicoGreen dye**. Negative is colorless, positive is showing green fluorescence and with the increase in dilution the green fluorescence is decreasing. The green fluorescence was detectable up to 9^th^ dilution and therefore the limit of detection was up to 53.4 copies.

**Fig. 4:**
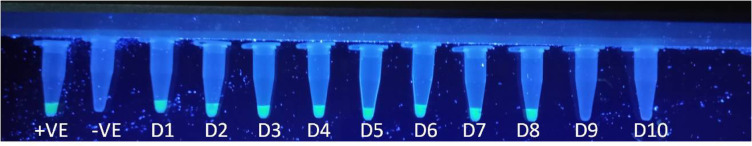
**Visual detection of posavirus PSR amplicons with different dilutions of plasmid DNA (10**^**−1**^ **to 10**^**–10**^**) using PicoGreen dye**. Negative is colorless, positive is showing green fluorescence and with the increase in dilution green fluorescence is decreasing. The green fluorescence was detectable up to 8^th^ dilution and therefore the limit of detection was up to 650.8 copies.

For the RPA assay, sensitivity was determined through visual inspection, with green fluorescence detectable up to the 9^th^ dilution, corresponding to a limit of detection of approximately 53.4 copies (Fig. 3). In the PSR assay, visual sensitivity revealed green fluorescence detectable up to the 8^th^ dilution, with a limit of detection of approximately 650.8 copies (Fig. 4).

### Analytical specificity of assays

**Fig. 5:**
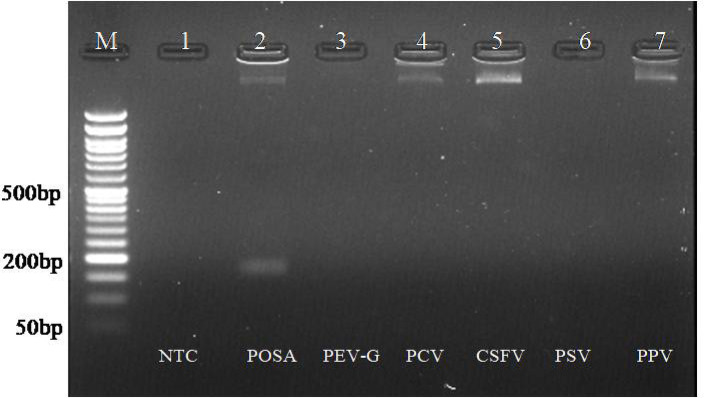
Analytical specificity of RPA assay. Lane M: 50 bp DNA ladder. L1: NTC, L2: RPA of posavirus with optimized conditions. L3-L7: Heterologous reaction using nucleic from PEV-G, PSV, PPV, PCV, CSFV respectively. Amplification only observed in Lane 2 having nucleic acid from posavirus.

**Fig. 6:**
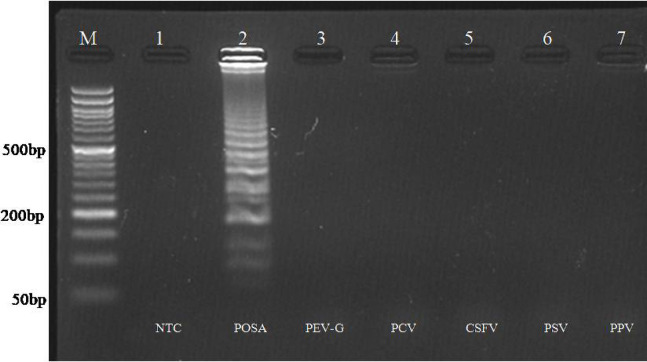
Analytical specificity of PSR assay. Lane M: 50 bp DNA ladder. L1: NTC, L2: PSR of posavirus with optimized conditions. L3-L7: Heterologous reaction with nucleic acid from PEV-G, PSV, PPV, PCV, CSFV respectively. Amplification only observed in Lane 2 having nucleic acid from posavirus.

To determine the specificity of both assays, it was tested with other related viruses from porcine, including classical swine fever virus, porcine enterovirus-G, porcine sapelovirus, porcine parvovirus, and porcine circovirus. Amplification was observed only with the porcine posavirus positive control, while no amplification occurred with nucleic acid derived from other viruses (Figs. 5 and 6).

### Evaluation of developed assay

The developed assays were validated using 132 field samples collected from various piggery farms. These samples were tested with developed RPA and PSR assay along with the conventional RT-PCR assays (Figure S10).

Three samples were found positive with developed RPA, PSR and with conventional RT-PCR assays by resolution under AGE. For visual detection of assay, picogreen dye was added in the posavirus PSR products and fluorescence was observed. The positive control showed fluorescence whereas negative control showed no fluorescence. However, three of the field samples displayed fluorescence, corroborating with the AGE results. Sequencing of one of the positive sample confirmed that it clustered within posavirus lineage 1, showing high nucleotide similarity with reference sequences previously reported from Asia and Africa^15^.

## Discussion

Diarrhea in pigs is a widespread and significant issue globally, leading to substantial financial losses for affected farms due to increased piglet mortality, diminished performance, and the costs related to treatment and control. Numerous studies have explored the swine intestinal virome as a potential contributing factor^3^. In addition to well-known astroviruses responsible for diarrhea, metagenomic next-generation sequencing (NGS) studies have identified several novel viral species whose biological roles in diarrhea development remain largely unexplored^10,23,24^. A study highlighted the potential of multiplex NGS workflows for investigating unexplained diarrhea in piglets, revealing increased viral loads in samples from problematic farms, particularly with read counts related to the viral genera Posavirus-1 and PoAstV-4 from Western Europe^12^.

For the development and optimization of the RPA and PSR assays for the posavirus, primers were in-house designed to target the polyprotein region of the genome. The optimization of the RPA assay indicated that a reaction temperature of 45 °C was ideal, resulting in the highest amplification of the target product. Previous studies have similarly demonstrated optimal RPA conditions that optimized an RPA-LFD assay for *D. graminicola* with temperatures ranging from 35 °C to 45 °C^25^.The reaction time for the posavirus RPA assay was optimized between 5 and 30 min, with 20 min identified as the ideal duration based on band intensity observed under agarose gel electrophoresis. This aligns with previous findings, where optimal reaction times for RPA-LFD assays developed for *Vibrio vulnificus*^26^ and *D. graminicola*^25^ were also established at 20 min.

Primer concentrations for the posavirus RPA were optimized within a range of 0.24 µM to 0.96 µM, with optimal values found to be 0.96 µM for the forward primer and 0.72 µM for the reverse primer at 45 °C for 20 min. This aligns with the primer concentrations optimized for porcine sapelovirus, where the forward primer was also set at 0.96 µM and the reverse primer at 0.48 µM^27^.These results highlight that it is crucial to adjust primer concentrations in order to improve test sensitivity and specificity. Additionally, the optimal concentration of magnesium acetate (MgOAc) for virus detection was found to be 16 mM, with the range of 10 mM to 20 mM being explored. Amplification was not significantly improved by higher concentrations, up to 38 mM. This is in line with other studies that showed that the ideal MgOAc concentrations ranged from 12 to 20 mM, with final doses of 14 mM^28^and 18 mM^29^ being particularly suggested for a variety of RPA tests that target distinct viruses.

The RPA assay for the posavirus exhibited a limit of detection of 5.34 × 10⁶ copies, detectable up to the fourth dilution, while conventional PCR demonstrated higher sensitivity, detecting up to 5.34 × 10³ copies at the eighth dilution. This indicates that the developed RPA assay is less sensitive than conventional PCR for detecting the posavirus. Indeed, earlier studies characterized RPA-LFD^30^ and RT-RPA^31^ assays as being approximately 10-fold less sensitive than real-time PCR assays.

The PSR assay for the posavirus was most effective at 66 °C; however, amplification products were observed across all tested temperatures, displaying remarkable results on agarose gel electrophoresis. Previous research has indicated rapid detection capabilities for *Mycobacterium avium* subsp. *Paratuberculosis*^32^ and *Salmonella* sp^33^ using temperature ranges of 61 °C to 69 °C, with optimal temperatures reported at 65 °C and 63 °C, respectively. In previous study, for optimization of RT-PSR have been done for detection of New Castle disease virus at 65℃ for 2 h^34^. Primer optimization for the posavirus PSR ranged from 1 µM to 4 µM, with final concentrations set at 4 µM for the forward primer and 3 µM for the reverse primer. These concentrations are comparable to findings for SARS-CoV-2^35^ and West Nile virus^36^ where the optimized concentrations were 1.6 µM and 4 µM, respectively. The limit of detection for the posavirus PSR assay was determined to be 6.5 × 10³ copies, exceeding the conventional PCR LOD of 6.5 × 10⁵ copies and underscoring the higher sensitivity of the PSR in this study. Earlier investigations indicated that PSR assays have been 100-fold or more sensitive for detecting West Nile virus nucleic acid compared to real-time PCR^36^.

Interestingly, while the RPA and PSR assays for the posavirus detected amplification products up to the fourth dilution on agarose gel, fluorescence changes using picogreen dye extended the detection limits to the ninth dilution for RPA and the eighth dilution for PSR. This observation corroborates findings from previous studies that demonstrated high sensitivity with dye-based detection methods^36–38^.

The specificity of the assays was evaluated by testing the posavirus RPA and PSR primers against related viruses, including PEV-G, PSV, PCV, PPV, and CSFV. The developed assays exclusively detected the posavirus genome without amplifying the genomes of other related viruses, demonstrating good specificity consistent with findings from similar studies targeting specific pathogens^39^. A total of 132 field samples were screened for the posavirus using two-step RT-RPA, RT-PSR, and conventional RT-PCR assays, where only three samples were tested positive for the posavirus.

Overall, the developed RPA and PSR assays represent promising alternatives for detecting the posavirus, with distinct advantages in terms of rapidity and ease of use^40,41^. Compared to one-step RT-PCR, both assays have several advantages. First, they operate at a constant low temperature, eliminating the need for expensive thermocyclers and can be performed with simple heat sources. Second, these assays are much faster, often completing within 20–60 min, whereas RT-PCR requires 1.5–2 h. Third, both isothermal assays exhibit high tolerance to inhibitors generally present in crude samples, reducing the need for extensive nucleic acid purification. Furthermore, their simplicity and rapid turnaround make them well suited for field diagnostics and resource-limited settings representing promising alternatives to one-step RT-PCR for rapid, cost-effective, and on-site pathogen detection. Nonetheless, further validation with a broader range of samples and comparative evaluations with established diagnostic methods are essential to confirm their utility in field applications^41^.

The current study combines the creation of quick diagnostic tests with our previous NGS finding of posavirus in Indian pigs. The discovery validates the virus’s existence in nearby herds and emphasizes the necessity for more extensive prevalence assessments, even though only three of the 132 field samples examined in this study tested positive. These clinically positive animals had moderate diarrhea, which is consistent with studies worldwide that posavirus is frequently found in both healthy and diarrheal pigs without a definite cause. Therefore, our work is a significant first step in connecting the identification of the virus in India with the development of diagnostic instruments necessary for upcoming outbreak investigations and prevalence studies.

## Conclusions

This study successfully developed and optimized two isothermal amplification assays, RPA and PSR, for the detection of the posavirus, providing reliable, rapid, and cost-effective alternatives to conventional PCR methods. Both tests showed good sensitivity and specificity, however the PSR assay was more sensitive. The diagnostic procedure was further expedited by the inclusion of Picogreen^®^ dye for visual detection, which improved the assays’ applicability for field use. Despite the fact that three of the analyzed field samples tested positive for the posavirus, these diagnostic methods offer a great deal of potential to help sustain ongoing monitoring and early detection initiatives, which will eventually result in improved disease management and control in the swine industry. Though this study indicates the presence of posavirus in Indian pig populations, it did not attempt to quantify prevalence, its clinical relevance or economic impact but rather provides the essential diagnostic foundation that will enable such epidemiological studies in India. Future research should focus on applying these methods in various epidemiological contexts to validate their effectiveness and utility.

## Supplementary Information

Below is the link to the electronic supplementary material.

Supplementary Material 1

## Data Availability

All data generated or analysed during this study are included in this published article and its supplementary information files.
