# Supplementary material for: Isothermal nucleic acid amplification assays for the detection of porcine stool-associated RNA virus
Source: Sci Rep. 2025 Oct 31;15:38196. doi: 10.1038/s41598-025-22146-4 (PMC12578852; doi:10.1038/s41598-025-22146-4)
Supplement: Supplementary file 1 — Supplementary Material 1 [file 41598_2025_22146_MOESM1_ESM.docx]

**Supplementary files for the manuscript entitled**

**Isothermal Nucleic acid Amplification Assays for the Detection of Porcine Stool-Associated RNA virus**

**Sarishti Kaushik, Sushila Maan*, Kanisht Batra,Swati Sindhu, Vijay Kadian and Aman Kumar**


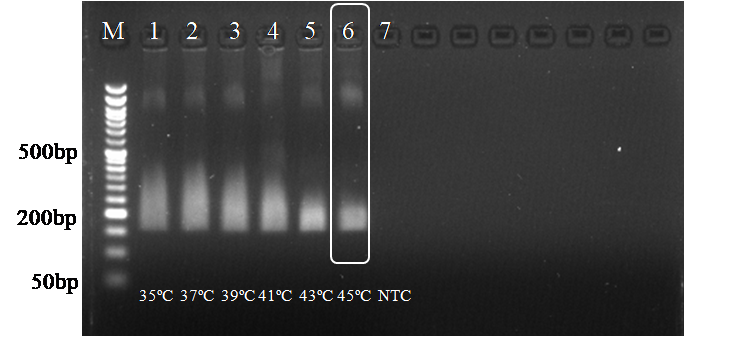


Figure S1: Agarose gel electrophoresis of posavirus RPA amplified product at variable temperatures.

M: 50 bp ladder, L1-L6: Posavirus gene 2 RPA at different temperatures 35ºC, 37ºC, 39ºC, 41ºC, 43ºC, 45ºC,L7: NTC.The final optimized temperature for RPA was 45ºC.


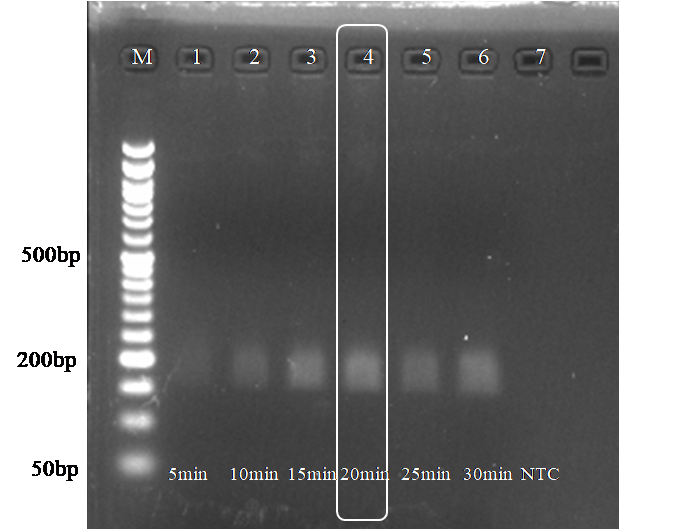


Figure S2: Agarose gel electrophoresis of posavirus RPA amplified product at variable time. M: 50 bp ladder, L7 : NTC, L1-L6 : RPA at different time 5 min., 10 min., 15 min., 20 min., 25 min., 30 min. The final optimized time for posavirus RPA was 20 min.


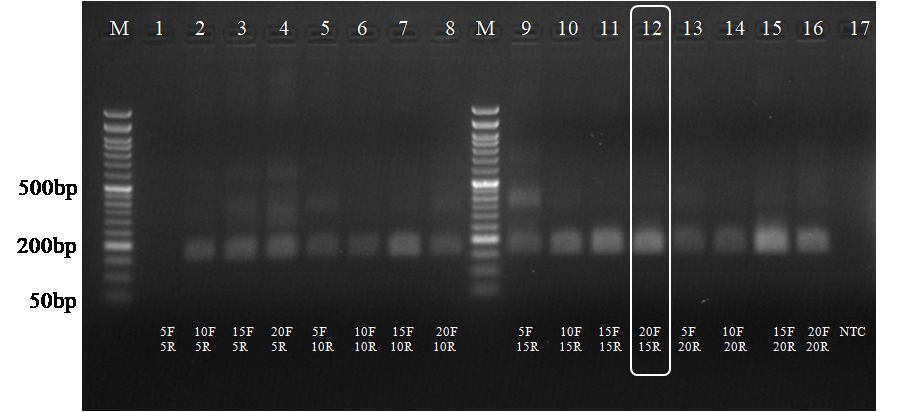


## Figure S3: Agarose gel electrophoresis of posavirus RPA optimization of RPA reaction for primer concentration on 2.5% gel. Lane M: 50 bp ladder, L17: NTC, L1: 5F5R, L2: 10F5R, L3: 15F5R, L4: 20F5R, L5: 5F10R, L6: 10F10R, L7: 15F10R, L8: 20F10R, L9: 5F15R, L10: 10F15R, L11: 15F15R, L12: 20F15R, L13: 5F20R, L14: 10F20R, L15: 15F20R, L16: 20F20R. Final optimized primer concentration for posavirus RPA reaction is 20F15R i.e 0.96μM of F primer and 0.72μM of R primer.

##
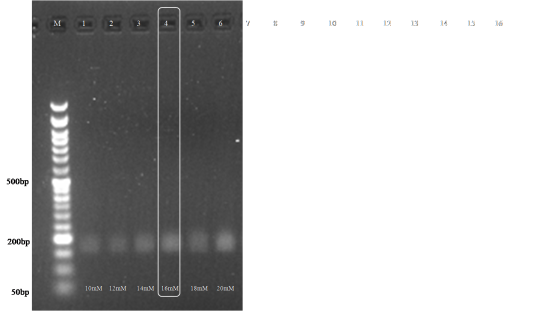


Figure S4: Agarose gel electrophoresis of optimization of posavirus RPA for MgOAc concentration on 2.5% gel. M: 50bp ladder, L1: 10mM, L2: 12mM, L3: 14mM, L4: 16mM, L5: 18mM, L6: 20mM. The final optimized MgOAc concentration was 16mM.

**
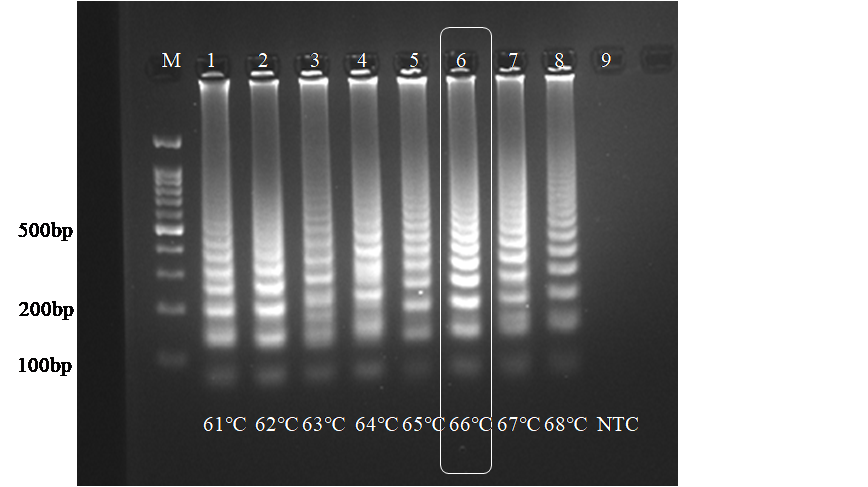
**

Figure S5: Agarose gel electrophoresis of posavirus PSR amplified product at variable temperatures.Lane M: 100 bp DNA marker; Lane 9: NTC; Lane 1-8: PSR amplified gene product at variable temperature (61℃, 62℃, 63℃, 64℃, 65℃, 66℃, 67℃ and 68℃)**.**The final optimized temperature for PSR was 66℃.


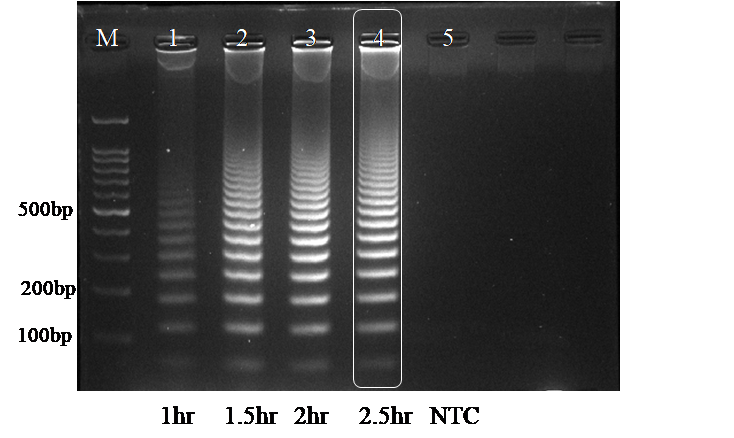


Figure S6: Agarose gel electrophoresis of posavirus PSR amplified product at variabletime. Lane M: 100bp DNA marker; Lane 5: NTC; Lane 1-4: PSR amplified gene product at variable time 1hr, 1.5hr, 2hr, 2.5hr. The final optimized time for posavirus PSR was 2.5hr.Thefinal optimized time for posavirus PSR was 2.5hrs.


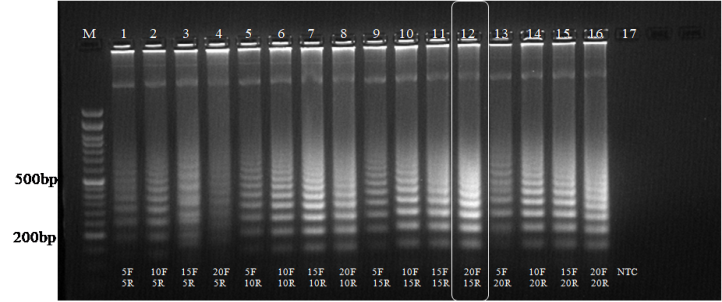


Figure S7: Agarose gel electrophoresis of posavirus PSR reaction for primer concentration on 2.5% gel. Lane M: 50bp ladder, L17: NTC, L1: 5F5R, L2: 10F5R, L3: 15F5R, L4: 20F5R, L5: 5F10R, L6: 10F10R, L7: 15F10R, L8: 20F10R, L9: 5F15R, L10: 10F15R, L11: 15F15R, L12: 20F15R, L13: 5F20R, L14: 10F20R, L15: 15F20R, L16: 20F20R.Final optimized primer concentration for posavirus PSR reaction is 20F15Ri.e.4μM of F primer and 3μM of R primer.


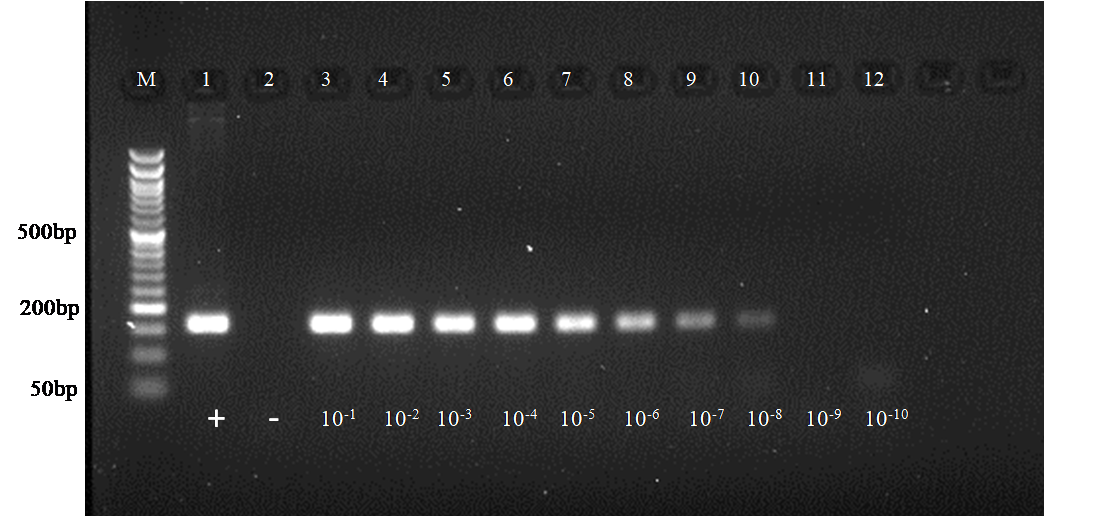


## Figure S8: Agarose gel electrophoresis of PCR reaction with posavirus plasmid (using RPA primers) dilutions on 2.5% gel. Lane M: 50bp ladder, L2: NTC. L1: Positive control. L3-L12: Serial 10-fold dilution of posavirus plasmid DNA (10^-1^ to 10^-10^). The assay could detect plasmid DNA upto 10^-8^ dilution.


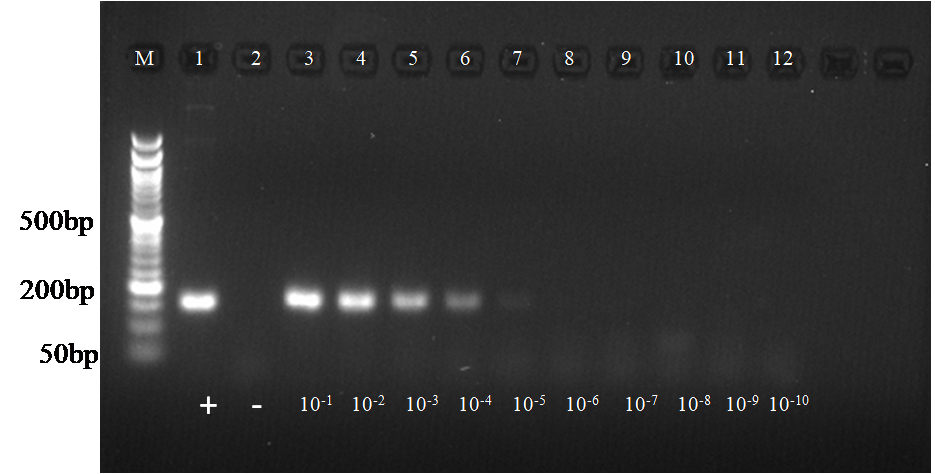


Figure S9: Agarose gel electrophoresis of posavirus PCR reaction with plasmid (using PSR primers) dilutions on 2.5% gel. Lane M: 50bp ladder, L2: NTC. L1: Positive control. L3-L12: Serial 10-fold dilution of posavirus plasmid DNA (10^-1^ to 10^-10^).The assay could detect plasmid DNA upto 10^-5^dilution.

**Figure S10:** Agarose gel electrophoresis of posavirus PCR reaction of three samples with positive control (using RPA primers) on 2.5% gel. Lane M: 50bp ladder, Lane 1: Negative control, L2: Positive control. L4-L6: Field samples positive for Posa virus. L7-14: Negative field samples.
